# Post Varicella-Zoster virus transverse myelitis: Diagnostic and therapeutic challenges – A case report and literature review

**DOI:** 10.1016/j.idcr.2025.e02336

**Published:** 2025-07-29

**Authors:** Mohammad Mehdi Shadravan, Farnoosh Farshchian, Alireza Rajaei, Ilad Alavi Darazam, Reza Naseri, Faezeh Maghsudloo

**Affiliations:** a Student Research Committee, Shahid Beheshti University of Medical Sciences, Tehran, Iran; b Department of Rheumatology, Loghman Hakim Hospital, Shahid Beheshti University of Medical Sciences, Tehran, Iran; c Department of Infectious Diseases and Tropical Medicine, Loghman Hakim Hospital, Shahid Beheshti University of Medical Sciences, Tehran, Iran; d Department of Radiology, Loghman Hakim Hospital, Shahid Beheshti University of Medical Sciences, Tehran, Iran; e Department of Neurology, Loghman Hakim Hospital, Shahid Beheshti University of Medical Sciences, Tehran, Iran

**Keywords:** Transverse myelitis, Varicella zoster virus infection, Systemic lupus erythematosus, Neuromyelitis optica, Case report

## Abstract

**Introduction:**

Acute transverse myelitis (ATM) is a rare inflammatory disorder that affects the spinal cord, leading to sudden weakness, sensory deficits, and bowel/bladder dysfunction. Also rare, this condition can be caused by infections such as the Varicella-zoster virus (VZV) or can occur as a complication of systemic lupus erythematosus (SLE). It has been reported to be more prevalent in SLE patients compared to VZV infections. We present a case of a patient with a history of SLE and evidence of vesicular rash from VZV infection.

**Case report:**

A 61-year-old female presented with progressive weakness in her lower limbs. Two weeks before, she had developed a vesicular rash due to a VZV infection in the T6-T9 dermatomes, which was followed by paraparesis, sensory loss, and urinary retention. She also had a history of SLE. During the physical examination, muscle strength and sensation were decreased in the lower limbs. MRI revealed central myelopathy from T6 to T10. In laboratory tests, VZV PCR was positive, and Aquaporin-4 was also negative. The patient was treated with IV corticosteroid pulse and ganciclovir, followed by plasma exchange. resulted in partial recovery.

**Conclusions:**

This case highlights VZV-induced TM (VZV-TM) in an immunocompromised patient with underlying SLE. Despite overlapping etiologies, a thorough clinical, radiologic, and laboratory evaluation, including a positive CSF VZV PCR and the absence of a SLE flare, supported VZV-TM as the final diagnosis. Prompt antiviral therapy and escalation to plasma exchange led to substantial neurological recovery.

## Introduction

Acute transverse myelitis (ATM) is a rare and focal inflammatory disorder that affects the spinal cord and usually causes sudden weakness, sensory deficits, and bowel or also bladder dysfunction. The disorder is typically a condition that develops on its own, sometimes as a result of an infection, but it can also be part of a range of other neuro-inflammatory disorders. The pathophysiology of ATM varies based on the specific etiology, making a thorough clinical assessment and investigation crucial for accurate diagnosis and appropriate management guidance [1], [2]. TM can be caused by Varicella-zoster virus (VZV); however, it is an uncommon condition that represents only 0.3 % of neurological disorders linked with the virus [3]. Furthermore, TM related to systemic lupus erythematosus (SLE-TM) is a severe complication of SLE that can significantly reduce quality of life. Although rare, it affects between 0.5 % and 1 % of all SLE patients and may present as the initial symptom in 30–60 % of these patients [4].

This is a case report of a patient who has been diagnosed with SLE for 30 years and had a VZV infection two weeks before admission to the emergency department with manifestations of TM. This case poses a significant clinical challenge in determining this patient's underlying cause of TM, which could be either SLE or VZV infection. This case report was based on the CAse REport (CARE) guidelines [5].

## Case report

A 61-year-old female presented to the emergency department with a chief complaint of progressive lower limbs weakness. Two weeks prior to admission, she had developed a truncal rash with a vesicular appearance over the left anterior (T6–T9 dermatomes) region, which was clinically diagnosed as varicella zoster infection by a general practitioner. Oral acyclovir (200 mg, three times daily) was prescribed at that time. Approximately ten days after the onset of the rash — that is, four days before hospital admission — the patient began to experience bilateral lower limb numbness, followed shortly by progressive weakness. These symptoms gradually worsened, and eventually, she developed urinary retention.

She had a medical history of SLE (from 30 years ago), endocarditis (Libman-Sacks), and a cerebral vascular accident. Her past medication history included prednisolone, hydroxychloroquine, warfarin, captopril, and mycophenolate mofetil. At the time of presentation, she was receiving prednisolone 5 mg daily and mycophenolate mofetil 1 g twice daily, with no recent changes in dosage. She had not received shingles (herpes zoster) vaccination prior to symptom onset.

On physical examination, she had a low-grade fever (37.5 °C). Her blood pressure was 160/80 mmHg, respiratory rate was 12/min, and pulse rate was 80/min. She had a vesicular rash in the left T6 to T9 dermatome. Consciousness, mental status, cranial nerves, and cerebellar testing were normal. Muscular motor power was normal in the upper extremities but decreased in both lower extremities (Medical Research Council (MRC) grade 2/5). The tone of the muscles was normal. Deep tendon reflex response scored 2 + in lower extremities. A diminished sensation of pain and light touch was noted. Sensory level in the T10 dermatome and Babinski sign was found. She was not able to walk.

**Fig. 1:**
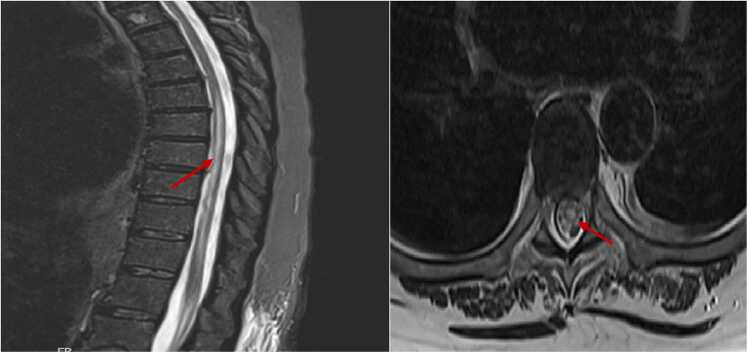
Sagittal (A) and axial (B) T2-weighted MRI of the thoracic spine demonstrate LETM. In panel A, the hyperintense signal (indicated by arrow) spans from T6 to T10 vertebral levels, consistent with a long-segment central cord lesion. Panel B shows a corresponding axial view with central cord hyperintensity, without evidence of significant cord swelling or mass effect.

Magnetic resonance imaging (MRI) of the thoracic spine demonstrated a longitudinally extensive T2-hyperintense lesion from T6 to T10 with contrast enhancement but without significant cord swelling or mass effect, consistent with inflammatory myelopathy. MRI of the cervical spine and brain showed no abnormalities (Fig. 1).

Laboratory tests revealed bi-cytopenia (White blood cell=2500/mL, Platelets=110000/mL), elevated erythrocyte sedimentation rate, and C-reactive protein (ESR=21 mm/h, CRP=56.3 mg/dL). viral serologies (Hepatitis B and C, Human immunodeficiency virus, Cytomegalovirus, and Herpes simplex virus) were negative. Βeta-2-glycoprotein Anti-dsDNA, anti-sjogren-related antigen A autoantibody, and Lupus anticoagulant were elevated. (Βeta-2-glycoprotein=6.9, Anti-dsDNA=131, Anti-Ro=47.3, Lupus anticoagulant=66). Vitamin B12 and folate were normal. Aquaporin-4 (neuromyelitis optica antibody) was negative.

Cerebrospinal fluid (CSF) analysis revealed an opening pressure of 120 mmH2O, red blood cell count of 500/μL, and marked lymphomonocytic pleocytosis with a white blood cell count of 1100/μL (60 % monocytes). Biochemical studies showed elevated protein (122 mg/dL), normal glucose (96–97 mg/dL), and elevated lactate dehydrogenase (LDH: 295 IU/L). VZV DNA was detected in the CSF by polymerase chain reaction (PCR), while HSV PCR was negative. Serum VZV IgG level was borderline (9.3), and CSF-specific IgG, IgG index, and oligoclonal bands were not assessed.

From these data, the diagnosis of VZV-induced transverse myelitis (VZV-TM) was made. The patient was treated with intravenous (IV) Methylprednisolone pulse therapy (1 g/day for six days) and IV ganciclovir two times a day, for a total of 14 days. Given the patient's immunocompromised status and the clinical suspicion for both VZV and cytomegalovirus (CMV), ganciclovir was preferred over acyclovir to ensure broader antiviral coverage during the early treatment phase. Despite a remote history of lupus nephritis, her renal function was within normal limits both at presentation and throughout the antiviral treatment course, with no evidence of ganciclovir-associated nephrotoxicity.

These treatments did not cause any clinical improvement. Thus, a five-session course of plasma exchange was performed, which caused a partial recovery. Upon discharge, strength was MRC grade 4/5 in the lower extremities and she was able to walk again. Two months after discharge, the patient regained full lower limb strength (MRC 5/5), was able to walk independently, and showed no signs of VZV reactivation or new neurological symptoms.

## Discussion

**Fig. 2:**
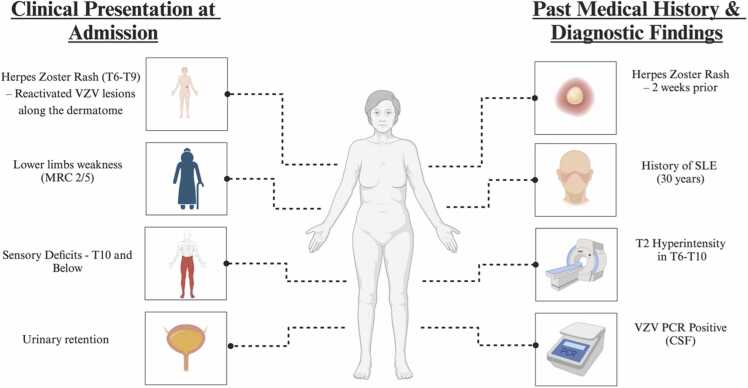
Summary of the patient’s clinical presentation at admission, past medical history, and key diagnostic findings.

Transverse myelitis is a rare complication of both SLE and VZV infection. A retrospective cohort analysis conducted by Dr. Costallat et al. examined 1193 patients diagnosed with SLE, with a focus on exploring different facets of myelopathy within this cohort. The findings revealed that myelopathy occurred in 1.2 % of SLE patients included in the study. Remarkably, this manifestation may serve as the initial presentation of the disease, occurring irrespective of the systemic activity of SLE. In cases where these two etiologies (VZV and SLE) are simultaneously plausible, recent literature provides helpful distinguishing features that can guide diagnostic reasoning [6], [7]. In a comprehensive review by Baisya et al., lupus myelitis is emphasized as a diagnosis supported primarily by evidence of active systemic disease, including elevated anti-dsDNA antibodies, reduced complement levels, and a high SLE Disease Activity Index score. Notably, lupus myelitis may present with either flaccid or spastic paralysis depending on whether the grey or white matter is predominantly involved, and nearly 40 % of patients may have a normal spinal MRI [8]. In contrast, Hung et al. retrospectively examined the characteristics of VZV-TM and found that patients typically present with a dermatomal vesicular rash followed by neurological symptoms within 2 weeks. Most patients demonstrated CSF pleocytosis, elevated protein levels, and normal glucose levels, with VZV DNA detectable in 76.5 % of CSF samples by PCR. MRI findings frequently showed longitudinally extensive lesions, predominantly in the thoracic cord [9]. Our patient showed no clinical or laboratory evidence of an SLE flare at the time of presentation. She had no systemic symptoms such as rash, arthritis, or serositis, and her immunosuppressive regimen had remained stable. While anti-dsDNA and antiphospholipid antibodies were mildly elevated, this can occur even in patients with inactive SLE, and complement levels were not significantly depressed. Notably, she developed a vesicular rash in the T6–T9 dermatome several days prior to the onset of neurological symptoms, and MRI demonstrated longitudinally extensive myelopathy from T6 to T10, anatomically overlapping with the dermatomes involved in the preceding vesicular eruption. A positive VZV PCR in the CSF further supported the diagnosis of active VZV infection. Although serum VZV IgG was borderline and CSF IgG was not assessed, PCR is known to be highly specific and most reliable in the first 7–10 days of CNS involvement. Considering these clinical, radiologic, and virologic findings—and despite the higher prevalence of TM in SLE patients—VZV was favored as the most probable etiology in our case. Nevertheless, the initial treatment approach included IV corticosteroids and ganciclovir to provide early and comprehensive coverage for both potential etiologies while awaiting confirmatory results [10], [11], [12], [13], [14]. Fig. 2^1^ provides a visual summary of the patient's clinical presentation, past medical history, and key diagnostic findings.

Regarding the choice of ganciclovir, although CMV-induced transverse myelitis is rare, it remains an important differential diagnosis—particularly in immunosuppressed patients. Prior reports have described CMV-associated myelitis with neurological features closely resembling those caused by VZV [15], [16]. Therefore, ganciclovir was initially chosen to provide antiviral coverage for both CMV and VZV while awaiting definitive PCR results.

**Fig. 3:**
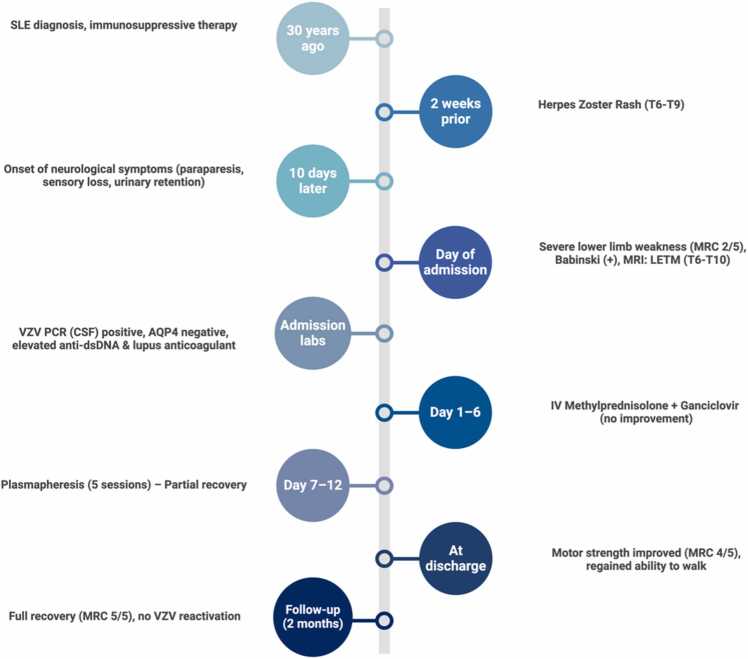
Timeline of clinical events.

After the positive result of VZV PCR, the final diagnosis (TM-VZV) was determined. Fig. 3^2^ illustrates the chronological progression of the patient's symptoms, diagnostic findings, and treatment response.

After reviewing the PubMed and Google Scholar databases, we have analyzed the reported cases of this uncommon manifestation in VZV-infected patients from 2020 to July 2024, as shown in Table 1 [3], [17], [18], [19], [20], [21], [22], [23], [24], [25], [26], [27], [28], [29], [30], [31], [32]. Our review of VZV-TM cases highlights diverse presentations across immune statuses, with generally good outcomes in immunocompetent patients. Notably, even some immunocompromised individuals, like our patient, had favorable responses, emphasizing that immune status alone is not a consistent prognostic factor.

**Table 1:** A summary of the clinical details of varicella-zoster virus myelitis case reports from 2020 until July 2024.

N	Year	Sex	Age	On admission			History of VZV infection		Zoster level	MRI level	Immunity	Treatments	Outcome	AQP4-Ab	R
				MW	S	E	Interval time	Diagnostic method							
1	2024	M	53	+	+	+	2 m prior	n.a.	n.a.	C2-C7, T3-conus	SARS-CoV2 infection / 2 m prior	ACV + S + IVIG + P + CTX	0	Negative	[17]
2	2023	M	74	+	–	+	1 w prior	+ VZV Ab serum, CSF	Generalized	T12-L1	I.C	ACV + S	0	n.a.	[18]
3	2023	F	68	+	+	+	3 w prior	+ PCR	T6-T9	T6-T9	COVID−19 vaccination / 4 w prior	ACV + S + GBP	1	Negative	[19]
4	2023	M	58	+	+	+	1 m prior	+ VZV Ab serum, CSF and PCR	Upper limb	C3-T2	I.C	ACV + S + IVIG	1	Negative	[20]
5	2023	F	27	+	+	+	23 d prior	n.a.	Generalized	Craniovertebral junction-C5, T2-T6	I.C	ACV + S	0	n.a.	[21]
6	2023	M	69	+	–	–	2 m prior	+ VZV Ab CSF	Scapula, Cubital region of upper limb	C3–4 to C7-T1	I.C	ACV	0	n.a.	[22]
7	2022	M	60	+	+	+	1 m prior	– PCR	T2-T4	C3-C4, T6	I.C	ACV + S + P	1	n.a.	[23]
8	2022	M	66	+	+	+	n.a.	+ VZV Ab serum, CSF	n.a.	T9-L5	DM	ACV + S	0	n.a.	[24]
9	2022	F	22	+	+	+	1 m prior	+ VZV Ab serum	Breast	C1-C4	I.C	S + P	0	Positive	[25]
10	2022	F	64	+	+	–	2 w prior	+ PCR	C4-T1	C1-C4	Immunocompromised	ACV + S	0	n.a.	[26]
11	2022	M	52	+	+	+	n.a.	+ VZV Ab CSF	Absent	T8-T11	I.C	GCV + S	0	n.a.	[27]
12	2021	M	42	+	–	+	10 d prior	+ RT-PCR	T8-T10	T4-T7, T11-L1	HIV	ACV + S	2	n.a.	[28]
13	2021	M	58	+	–	–	3 w prior	+ VZV Ab CSF	T4	C7-T4	I.C	ACV + S + IVIG + P	1	n.a.	[29]
14	2021	M	46	+	+	+	3 w prior	+ VZV Ab serum	T5-T8	T2–8	I.C	ACV + S	0	n.a.	[30]
15	2021	M	38	+	+	+	1 m prior	n.a.	n.a.	T3-T11	HIV	ACV + S + Vancomycin + Emergent T9–T12 laminectomy	1	n.a.	[31]
16	2021	M	28	+	+	+	10 d prior	+ VZV Ab serum and PCR	Generalized	Normal	I.C	ACV + S	0	n.a.	[3]
17	2020	M	63	+	+	+	2 w prior	+ VZV Ab serum and PCR	T5	Cervicomedullary junction-T9	I.C	ACV + S + P	0	n.a.	[32]

Abbreviations: N: Number; M: Male; F: Female; AQP4-Ab: Aquaporin-4 Antibody; MW: Motor Weakness; S: Sensory loss; E: Sphincter; m: Month; w: Week; d: Day; n.a: Not Available; VZV: Varicella-Zoster Virus; PCR: Polymerase Chain Reaction; RT-PCR: Reverse Transcription Polymerase Chain Reaction; CSF: Cerebrospinal Fluid; Ab: Antibody; MRI: Magnetic Resonance Imaging; I.C: Immunocompetent; DM: Diabetes Mellitus; HIV: Human Immunodeficiency Virus; ACV: Acyclovir; GCV: Ganciclovir; S: Steroids; P: Plasma Exchange; IVIG: Intravenous Immunoglobulin GBP: Gabapentin; CTX: Cyclophosphamide; 0: good outcome (ability to walk independently or with aid); 1: poor outcome (inability to walk even with aid); 2: death.

Hung et al.'s retrospective study (1980–2012) on VZV-TM revealed an association between patient immune status and disease outcome. Immunocompromised individuals were more susceptible to VZV infection-related transverse myelitis, exhibiting atypical presentations and poorer prognoses compared to immunocompetent patients. Subsequently, Sebastian et al.'s study (2001–2020) demonstrated that neurological complications could arise in both immunocompromised and immunocompetent hosts [3], [9]. According to Table 1, among the 17 cases reported from 2020, most of the patients were immunocompetent, and with acyclovir and steroid therapy (although the treatment was different in some cases), most of the patients responded well to the treatment and had a good outcome. However, despite being immunocompetent, some of these patients [20], [23], [29] had a weaker response to treatment. Also, based on the table, it can be seen that immunocompromised patients are more likely to lead to poor outcomes and even death of patients [28], [31]. However, being immunocompromised is not a definite reason for the bad prognosis of VZV-TM patients, and some patients have had a good outcome. However, our patient was immunocompromised but had a typical presentation and a good outcome.

The clinical presentation of VZV-TM patients can manifest in both typical and atypical forms. The typical presentation involves the sequential occurrence of skin lesions followed by myelopathy at the corresponding dermatomal level. However, atypical presentations are characterized by various deviations from this norm. These include myelopathy developing without preceding shingles, a condition known as zoster sine herpete, skin lesions appearing after the onset of myelopathy, and the discordance between the anatomical distribution of myelopathy and zoster lesions. In a study conducted by Hung et al., where 31 VZV-TM patients were examined, including 17 individuals with immunocompromised status, most notably due to AIDS, it was found that immunodeficiency significantly predisposes patients to atypical manifestations. Specifically, both permanently and transiently immunocompromised individuals exhibited a higher propensity for atypical presentations (p < 0.05) [9]. Our patient presented with typical clinical features, including vesicular rash in the T6-T9 dermatomes and myelopathy affecting the T6-T10 levels on MRI (Fig. 1).

Whenever feasible, the diagnosis of ATM should be supplanted by disease-specific terms to delineate the underlying etiology and inform management strategies. A thorough assessment of clinical, radiological, serological, and cerebrospinal fluid (CSF) characteristics aids in narrowing the spectrum of differential diagnoses and distinguishing mimics of ATM [2]. Florid and widespread spinal cord inflammation cause T2 hyperintensity on spinal magnetic resonance imaging that extends over three or more vertebral segments, defining longitudinally extensive transverse myelitis (LETM) [33]. Based on this, our patient, who had extensive involvement in T6-T9 segments, was identified as LETM.

LETM is an uncommon but potentially severe condition, and early identification of its underlying cause is crucial for timely treatment and optimal outcomes. Potential etiologies must be thoroughly considered in the differential diagnosis. Infectious etiologies such as tuberculosis and syphilis have been reported to cause LETM, although tuberculosis was ruled out in our patient with negative testing. Systemic autoimmune conditions, including neurosarcoidosis (CNS sarcoidosis) and Sjögren’s syndrome, can also present with longitudinally extensive myelitis. In addition, LETM may occur as a paraneoplastic phenomenon associated with underlying malignancy (for example, as a paraneoplastic myelitis in patients with cancer). A thorough workup to exclude these alternative etiologies is therefore essential when evaluating a patient with LETM. Although LETM has many differential diagnoses, it is classically associated with neuromyelitis optica spectrum disorder (NMOSD) [33], [34]. NMOSD are a type of autoimmune disease that primarily causes demyelination in the optic nerves and spinal cord. Asian and black patients are more prone to CNS involvement compared to white patients, who often experience the onset of symptoms around the age of 40 [35]. Recent studies propose a potential association between infectious events, particularly Varicella zoster virus (VZV), and NMOSD development [36]. Aquaporin-4 Immunoglobulin G (AQP4-IgG) is a crucial diagnostic biomarker for NMOSD, with a positive test result in over 80 % of patients. While it is crucial for differentiating NMOSD from multiple sclerosis, it is not a reliable method for monitoring disease activity or predicting relapse. Additional diagnostic tests encompass biomarkers such as neurofilament light chain (NfL) and glial fibrillary acidic protein (GFAP), which aid in distinguishing NMOSD from other CNS disorders and monitor the disease's progression. For this reason, we also performed an AQP4-IgG test for neuromyelitis optica in our patient, which was negative [37].

In 2022, Dubey et al. described a case involving a young woman who developed longitudinally extensive transverse myelitis with positive AQP4 antibody titers subsequent to varicella zoster infection. They proposed that damage to AQP4-rich spinal cord tissue was likely mediated by anti-AQP4-IgG antibodies following VZV infection [25].

Currently, there is no definitive cure for ATM, and available therapies aim to alleviate symptoms by reducing spinal cord inflammation and immune-mediated myelin destruction. Initial immunotherapy during the acute phase aims to halt disease progression. First-line treatments involve high-dose intravenous corticosteroids such as methylprednisolone or dexamethasone. Plasma exchange and intravenous immunoglobulin serve as second-line options for steroid-unresponsive patients. Sebastian et al. emphasize that administration of acyclovir and methylprednisolone may lead to improved prognosis among patients with VZV-TM [3], [38]. Regarding our patient, considering that there was no significant clinical improvement after receiving six days of IV Methylprednisolone pulse therapy and also ganciclovir, we started plasma treatment for the patient (for five sessions), which caused partial recovery.

In conclusion, this case highlights a rare but informative presentation of transverse myelitis (TM) in an immunosuppressed patient with long-standing SLE, ultimately attributed to VZV infection. The diagnostic challenge lay in distinguishing VZV-induced TM from SLE-related TM in a patient with overlapping risk factors and chronic immunosuppression. Clinical decision-making required early broad antiviral coverage with ganciclovir, followed by timely escalation to plasma exchange when initial therapies failed. Despite her immunocompromised state, the patient demonstrated a classic VZV-TM pattern and experienced full recovery. This case underscores the importance of early broad antiviral coverage, timely escalation to second-line therapies, and comprehensive evaluation of differential diagnoses—including NMOSD via AQP4 antibody testing. Ultimately, this case reinforces the need for personalized diagnostic reasoning and timely therapeutic decisions in complex neuroinfectious presentations.

## List of abbreviations

**ATM**: Acute Transverse Myelitis; LETM: Longitudinally Extensive Transverse Myelitis; SLE: Systemic Lupus Erythematosus; VZV: Varicella-Zoster Virus; HZ: Herpes Zoster; NMOSD: Neuromyelitis Optica Spectrum Disorder; AQP4: Aquaporin-4; PCR: Polymerase Chain Reaction; MRI: Magnetic Resonance Imaging; CSF: Cerebrospinal Fluid; MRC: Medical Research Council; ICP: Intracranial Pressure; ESR: Erythrocyte Sedimentation Rate; CRP: C-Reactive Protein; IgG: Immunoglobulin G; dsDNA: Double-stranded DNA; CNS: Central Nervous System; CVS: Cardiovascular System; HIV: Human Immunodeficiency Virus

## CRediT authorship contribution statement

**Mohammad Mehdi Shadravan:** Writing – review & editing, Writing – original draft. **Farnoosh Farshchian:** Writing – original draft. **Reza Naseri:** Writing – review & editing, Writing – original draft. **Faezeh Maghsudloo:** Writing – review & editing, Supervision, Project administration. **Alireza Rajaei:** Writing – review & editing. **Ilad Alavi Darazam:** Writing – review & editing, Supervision.

## Ethics approval and consent to participate

Ethical approval was not required for this case report in accordance with the policies of the Ethics Committee and Institutional Review Board of Shahid Beheshti University Medical Center, Tehran, Iran. Written informed consent was obtained from the patient for publication of the clinical data and images.

## Informed consent for publication

The patient gave written consent for the case to be published. All the authors have approved a copy of the consent form, which is available for review by the Editor of this journal.

## Role of the funding source

This research did not receive any specific grant from funding agencies in the public, commercial, or not-for-profit sectors.

## Declaration of Competing Interest

The authors declare that they have no known competing financial interests or personal relationships that could have appeared to influence the work reported in this paper.

## Data Availability

All relevant data from the clinical case are included in this article. Information on previously reviewed cases comes from publications available in the medical literature cited in the manuscript.

## Footnotes

1 Created in BioRender. Shadravan, M. (2025) https://BioRender.com/x63f575.

2 Created in BioRender. Shadravan, M. (2025) https://BioRender.com/b31y752.
